# Blended learning: ten tips on how to implement it into a curriculum in healthcare education

**DOI:** 10.3205/zma001338

**Published:** 2020-09-15

**Authors:** Inga Hege, Daniel Tolks, Martin Adler, Anja Härtl

**Affiliations:** 1 Universität Augsburg, Med. Fakultät, Lehrstuhl Medical Education Sciences, Augsburg, Germany; 2 Klinikum der LMU München, Institut für Didaktik und Ausbildungsforschung in der Medizin, München, Germany; 3 Leuphana Universität Lüneburg, Zentrum für Angewandte Gesundheitswissenschaften, Lüneburg, Germany; 4 Instruct gGmbH, München, Germany; 5 Universität Augsburg, Med. Fakultät, Lehrstuhl für Medizindidaktik und Ausbildungsforschung, DEMEDA, Augsburg, Germany

**Keywords:** Blended Learning, curriculum, curriculum development

## Abstract

Blended learning is a meaningful combination of online and face-to-face teaching and learning. In this article we summarize relevant aspects of this format and provide ten tips for educators and curriculum developers on implementing a blended learning curriculum in healthcare education. These general tips are derived from our experience and the available literature and cover the planning and implementation process.

## Zusammenfassung

Blended Learning ist eine sinnvolle Verknüpfung von Online- und Präsenzlehre. In diesem Artikel werden relevante Aspekte dieses Formats zusammengefasst und zehn Tipps zur Umsetzung eines Blended Learning-Curriculums in der Ausbildung in den Gesundheitsberufen gegeben. Die allgemeinen Tipps wurden sowohl aus der persönlichen Erfahrung der Autor*innen als auch auf Basis der verfügbaren Literatur abgeleitet und umfassen sowohl den Planungs- als auch den Umsetzungsprozess.

## What is blended learning?

An example of a well-known blended learning setting is the inverted or flipped classroom, in which students complete self-directed online activities to prepare for a subsequent face-to-face session that builds on the previously acquired knowledge [1]. Generally speaking, blended learning is a meaningful, aligned combination of online and face-to-face teaching and learning that takes advantage of both approaches.

There is no real consensus in the literature on how, in detail, a learning scenario should be designed in order to be called blended learning, and discussions often focus on the proportion and interaction of online and face-to-face teaching activities [2]. However, some elements of blended learning that enhance the understanding of the concept have been defined:

1. the learners have at least some control over when, where, and how they work,
2. technology is used to leverage personalization, and
3. instruction provides an integrated learning experience, i.e. the online and face-to-face instruction is aligned in a meaningful way [3].

## What are the advantages?

Some studies showed advantages of blended learning. A meta-analysis found that blended learning in the health professions seems to be more effective or at least as effective as traditional instruction for knowledge acquisition, but the heterogeneity of study settings and contexts makes conclusions difficult [4]. However, as blended learning bridges traditional face-to-face and online learning, it can reduce the shortcomings of both formats. For example, a lack of personal contact with peers and tutors in e-learning activities is compensated for by the face-to-face sessions, and a lack of self-paced learning time in face-to-face teaching is compensated for by the online environment.

Moreover, blended learning helps to bridge the gap between theory and practice and has the potential in healthcare education to improve learners’ clinical competencies, such as clinical reasoning or documentation skills [5].

## What are the disadvantages and barriers?

Despite such benefits and technological advancements, the integration of online learning into a healthcare curriculum in the form of aligned blended learning is still limited in Germany.

The reasons for and potential barriers to a more widespread uptake include, for example:

1. low familiarity of educators with online learning and blending,
2. initial effort, including costs, to create high-quality online learning material, and
3. concerns that students are not familiar with the format and will not complete the online material [6], [7].

## How can I develop and implement a blended learning curriculum?

In the following sections we propose ten general tips to help educators and curriculum designers to conceptualize and implement a blended learning curriculum in order to overcome barriers and make use of the advantages of blended learning. The tips are based on the literature on blended learning and our experience in implementing blended learning scenarios for the last 20 years. We, as authors, have different perspectives on blended learning making it possible to provide perspectives on curriculum development, e-learning, face-to-face teaching and faculty development in this article. Some of the tips may seem very basic, but often such obvious aspects are not sufficiently considered when planning blended learning scenarios. For each tip, we include examples or suggestions for further reading.

### 1. Apply the Kern cycle for curriculum development

The planning and implementation of a curriculum or the reform of a curriculum is often based on the six-step Kern cycle of curriculum development. This cycle includes

1. problem identification,
2. targeted needs assessment,
3. formulating goals and learning objectives,
4. selecting educational strategies,
5. implementation, and
6. evaluation and feedback [8].

Naturally, these steps are also useful for developing blended learning curricula and have been adapted by Chen et al. for online teaching [9]. The adapted cycle includes step-specific aspects for online teaching, such as the need for technological support for the implementation step or deciding about learning platforms when selecting suitable educational strategies. This adapted Kern cycle can serve as a helpful framework for the development of a blended learning course or curriculum, ensuring that no steps are omitted, and online and face-to-face phases are planned together and closely aligned.

Most importantly, we recommend planning the revision of the curriculum early, whether it be after evaluation results are available or even during the evaluation process (step 6). Often, curriculum development stops here and does not continue the cycle of refining and improving. Nevertheless, especially when developing a new curriculum with a novel format, planning the revision phases is essential.

#### 2. Apply an instructional framework for the design

A common approach for developing blended learning is converting traditional courses by simply adding some technology-based learning activities, often resulting in extra workload for teachers and especially students [10]. Instead, we recommend re-designing the entire curriculum or course and applying an instructional framework for the design of the online and the face-to-face phases. For example, Merrill et al. introduced five principles of instruction that can be applied to blended learning and can especially help to design state-of-the-art, face-to-face and online phases that actively engage the learner [11].

The five principles include

1. problem-centeredness with real-world problems,
2. learner activation,
3. demonstration of new knowledge or skills for the learner,
4. application of new knowledge or skills by the learner, and
5. integration with synthesis and reflection.

Often, online activities do not include the application and integration level, despite this being technically possible and a much more effective use of the online activities. For this reason, we recommend putting a special emphasis on these steps, for example, by engaging learners in collaboratively developing their own examples or cases in an online environment which then can be presented and discussed in a seminar. 

#### 3. Train the faculty on blended learning and build a team

Faculty training on how to develop and implement blended learning and how to discuss their new role [2] in such a student-centered approach is essential and can be a good starting point for building a development team.

We recommend familiarizing the faculty with the new approach by teaching them in a longitudinal, blended learning format including follow-up sessions, which then can nicely blend into the development process and educators can build upon their own experience as learners [12]. A fundamental aspect for faculty training is to use the same platforms, tools, frameworks, and settings that are used for teaching the students.

When starting, it is helpful to develop one or more pilot implementation as an example for your faculty.

Similar to a face-to-face curriculum, the development of a blended learning curriculum requires different perspectives and skills. Ideally, the team consists of a subject matter expert, an instructional designer and e-learning specialist, technical support, and an expert on face-to-face teaching working in close collaboration with other areas, such as assessment and evaluation. We admit that this might be difficult to achieve, but watching for faculty, staff or students with such experience and discussing your ideas and activities with them can help to build such a team in the long term.

#### 4. Involve the target group – you are not the learner

Part of the Kern cycle of curriculum development is identifying the needs of the intended target group, including learners with disabilities (steps 1 and 2). Therefore, we recommend involving your target group - the learners at a specific stage in their education who will experience your blended learning curriculum and the educators who will teach in this setting - in all steps of the curriculum development process. Since blended learning is also suitable to teach in interprofessional contexts [13], be aware to include all relevant target groups in such settings (e.g. medical, nursing and physiotherapy students). Learners can contribute their experiences with and approaches to learning in a rapidly changing technological environment, and they should be encouraged to share their own ideas.

The target group should also be involved in (pilot) testing activities and learning scenarios, and their feedback should be obtained as a basis for further refinement and development.

Such an approach can also be used to assess the digital competency of your learners at an early stage and enable you to address needs adequately.

For example, during the online phases students become familiar with formats such as e-portfolios, virtual patients, adaptive learning environments, or social media, which can be followed up on and discussed in the face-to-face sessions.

Another more challenging approach is to involve the target group as content creators by designing learning scenarios in which they are required to create content (e.g. a video clip) that can be re-used later on or for the next group of learners.

By involving the target group in multiple ways during the development process, it is more likely that you will meet the needs of your future learners and that they will accept the implemented formats.

#### 5. Clarify incentives for educators and legal aspects

When starting to plan and develop blended learning, educators often fear a loss of accredited teaching time while, at the same time, having to spend more time preparing material for the online phases. Unfortunately, there is no easy answer to this and, for example in Germany, universities and states have different rules for accrediting (online) teaching time [14].

As a consequence, we recommend having an open discussion about accreditation and finding solutions so that educators do not experience the disadvantages connected with blended learning. In any case, the online phases need to be an integral part of the teaching and included in the workload calculation for students and teachers.

Other topics that need to be addressed early on as part of steps 1 and 2 of the Kern cycle are data protection and copyright aspects concerning the online phases, especially in relation to feedback and learning analytics since access to learners’ activities and results are required. Recommended guidelines and resources include, for example, a copyright document provided by the Würzburg-Schweinfurt University of Applied Sciences [https://urheberrecht.fhws.de/faq-urheberrecht/], e-teaching.org [https://www.e-teaching.org/technik/datenhaltung/datenschutz] and a MOOC [https://imoox.at/mooc/local/courseintro/views/startpage.php?id=44] on data protection.

#### 6. Build on a stable and modern technical infrastructure 

To implement a blended learning curriculum (step 4 of the Kern cycle), it is essential to have a stable and accessible [https://www.e-teaching.org/didaktik/konzeption/barrierefreiheit] technical infrastructure for the learning environment in terms of hardware and software. According to the student association “Digital Changemaker” at the 2019 conference of the "Hochschulforum Digitalisierung", even basics like access to power and internet connections can be a challenge. This is nothing you can address yourself, but it is important to feed such information back to the faculty and university and to support your institution in implementing necessary changes for a reliable digital infrastructure. However, to avoid being overwhelmed by technical aspects, especially if there is no technical expert on your team, we recommend starting by assessing the learning environment available at your institution and building on what is already in use, even if this means compromising in the beginning. 

Even though open source software, such as the learning management system moodle [https://moodle.org/] or the e-portfolio platform mahara [https://mahara.org/], is available, it does need professional maintenance. Servers and software, in general, become quickly outdated without professional and regular service and can pose a security risk to users and the network. EU data protection regulations (GDPR) require a minimum security standard even if only minimal user information such as email addresses is stored. Consequently, the technical infrastructure needs to be maintained professionally, either by an institution's computer center or external services.

#### 7. Provide a common thread

A common thread is an overarching topic or guiding theme running through the curriculum. Providing one or more common threads for blended learning units and the overall curriculum helps both students and educators to navigate the curriculum and provides the "big picture" behind all online and face-to-face activities. The type of common thread might depend on the type of the curriculum, meaning it could be a problem, a cardinal symptom, a case, or a competency. In a spiral curriculum the common threads can be picked up again several times with increasing complexity and serve as a framework. For example, the cardinal symptom "cough" could serve as a common thread combining online teaching about the pathophysiology with face-to face bedside teaching and seminars addressing history taking and physical examination in patients with cough, virtual patients to train differential diagnoses, and students creating and presenting a concept map visualizing relevant aspects.

#### 8. Combine and align different teaching and assessment methods 

In general, the following combinations of online and face-to-face phases are possible:

1. online learning as preparation for face-to-face courses (so-called flipped or inverted classroom) and/or
2. online learning as a follow-up after a face-to-face course and/or
3. online elements during a face-to-face session, such as working in simulated environments or part-task trainers.

Depending on your learning objectives, there are many different formats and activities to choose from when developing the online and face-to-face phases; various websites provide guidance (e.g. [http://methodenpool.uni-koeln.de/frameset_uebersicht.htm], [https://fctl.ucf.edu/teaching-resources/teaching-strategies/teaching-methods-overview/]).

As part of step 4 (selecting educational strategies) in the Kern cycle, we recommend combining a variety of different teaching methods and settings to optimally meet the learning objectives you defined in step 3 of the Kern cycle. Just as for traditional curricula, learning objectives, teaching methods and formative and summative assessment should be aligned with each other, consistent with the model of constructive alignment [15].

Additionally, a variation of teaching methods can lead to a more satisfactory learning experience for the students [16].

For the online phases, we recommend not only aiming for obvious levels of competencies, such as knowledge, but also using activities to address higher-level learning objectives. For example, students can be engaged in online group activities to collaboratively solve a problem, which could then be presented and discussed in the face-to-face session.

As an example for combining formats in teaching clinical skills, videos and interactive online tasks can be used to prepare for a face-to-face course in which students train a clinical skill with simulated patients or part-task trainers; after the face-to-face course the trained skill can be put into a wider clinical context using virtual patients prior to bedside teaching rounds with patients in the hospital.

#### 9. Integrate open educational resources (OER)

Step 5 of the Kern cycle is implementation, which applies in blended learning scenarios to the face-to-face and the online teaching and the alignment of these phases.

Since online learning has been around for a long time, there is an abundance of material available for designing the online phase and reusing such material can save time. However, language might be a barrier to the re-use of existing high-quality material, which is often only available in English.

By integrating material from platforms that are already used by your learners, the barriers will be lower for them and they will not view the online components as something they have to do in addition or separately from what they already learn with. Introducing additional material and resources gives them a wider variety of available resources for their own learning. Resources and platforms such as YouTube [https://www.youtube.com], AMBOSS [https://www.amboss.com], or social media channels can be integrated, and learners can be engaged in identifying high-quality content (see tip 4). Plus, a critical reflection on the resources can be implemented in both the online and the face-to-face phases to improve students’ digital literacy skills.

Also, Massive Open Online Courses (MOOCs) can be integrated into a blended learning curriculum; de Jong et al. provide helpful tips on how to do this [17].

Finally, if you or your learners create material, consider publishing it as OER. To ensure that it is also suitable for other curricula, it can be helpful to develop material jointly in national or even international collaboration. Grants are often available for such collaboration and various helpful websites, such as OERInfo or OER InForm, are available to guide the creation and use of OERs [[https://open-educational-resources.de/], [https://oer.amh-ev.de/].

This tip addresses two barriers to introducing blended learning: the costs and the technological barriers for learners.

#### 10. Make full use of evaluation and feedback opportunities

For step 6 of the Kern cycle an evaluation framework, as described by Pombo et al. [18] or Bowyer et al. [19], can form the basis for holistically evaluating a blended learning curriculum instead of evaluating the online and face-to-face phases separately.

In addition to classic evaluation with questionnaires, the online phases provide cumulated and individual usage data (learning analytics). These data allow early identification of any issues with the course or learners and it may be possible to react during a course. The analysis of the usage data can also provide the basis for feedback and discussion in both the online and the face-to-face sessions.

As mentioned earlier, feedback (teacher-student or student-student) is an overarching principle for effective learning and should be present in online and face-to-face phases; feedback can also be integrated into and aligned with the evaluation activities.

Finally, we recommend setting up educational research studies based on your evaluations because there are still clear research gaps in how different types of learning influence academic performance, student content interactions, and the role of the teacher looking into both delivery modes [2].

## Conclusions

These ten tips represent the core of our experience with implementing blended learning curricula and courses. With the general tips we focused on the specifics of blended learning and only touched on some aspects that are relevant to online learning or face-to-face teaching in general. However, guidelines on how to implement the different phases are, of course, also valid and helpful in blended learning scenarios and should be considered. Implementing a blended learning curriculum is not about digitalizing learning content at all costs, but about taking advantage of online and face-to-face teaching.

Finally, be creative and have fun. Developing a blended learning curriculum is work, but it is also fun. You and your team can be creative in developing learning activities for both delivery modes and during the process you can learn a lot from the participating students and team members, each of whom has a different perspective on the curriculum.

## Competing interests

The authors declare that they have no competing interests. 
